# Developing Sustainable and Impactful Mobile Phone HIV Testing Interventions for Spanish-Speaking Men Who Have Sex With Men in the United States: Lessons Learned From Informative Interviews

**DOI:** 10.2196/publichealth.8992

**Published:** 2018-04-24

**Authors:** Jason Mitchell, Maria Beatriz Torres, Lucy Asmar, Thu Danh, Keith J Horvath

**Affiliations:** ^1^ Office of Public Health Studies University of Hawai'i at Mānoa Honolulu, HI United States; ^2^ Department of Communication Studies Gustavus Adolphus College St Peter, MN United States; ^3^ Department of Public Health Sciences University of Miami Miller School of Medicine Miami, FL United States; ^4^ Division of Epidemiology and Community Health School of Public Health University of Minnesota Minneapolis, MN United States

**Keywords:** smartphone, HIV testing, HIV prevention, men who have sex with men, Spanish

## Abstract

**Background:**

Although many men who have sex with men (MSM) test for HIV at least once in their lifetime, opportunities to improve regular HIV testing, particularly among Hispanic or Latino MSM, is needed. Many mHealth interventions in development, including the ones on HIV testing, have primarily focused on English-speaking white, black, and MSM of other races. To date, no studies have assessed app use, attitudes, and motivations for downloading and sustaining use of mobile apps and preferences with respect to HIV prevention among Spanish-speaking, Hispanic MSM in the United States.

**Objective:**

The primary aims of this study were to determine what features and functions of smartphone apps do Hispanic, Spanish-speaking MSM believe are associated with downloading apps to their smartphones, (2) what features and functions of smartphone apps are most likely to influence men’s sustained use of apps over time, and (3) what features and functions do men prefer in a smartphone app aimed to promote regular testing for HIV.

**Methods:**

Interviews (N=15) were conducted with a racially diverse group of sexually active, HIV-negative, Spanish-speaking, Hispanic MSM in Miami, Florida. Interviews were digitally recorded, transcribed verbatim, translated back to English, and de-identified for analysis. A constant-comparison method (ie, grounded theory coding) was employed to examine themes that emerged from the interviews.

**Results:**

Personal interest was the primary reason associated with whether men downloaded an app. Keeping personal information secure, cost, influence by peers and posted reviews, ease of use, and functionality affected whether they downloaded and used the app over time. Men also reported that entertainment value and frequency of updates influenced whether they kept and continued to use an app over time. There were 4 reasons why participants chose to delete an app—dislike, lack of use, cost, and lack of memory or space. Participants also shared their preferences for an app to encourage regular HIV testing by providing feedback on test reminders, tailored testing interval recommendations, HIV test locator, and monitoring of personal sexual behaviors.

**Conclusions:**

The features and functions of mobile apps that Spanish-speaking MSM in this study believed were associated with downloading and/or sustained engagement of an app generally reflected the priorities mentioned in an earlier study with English-speaking MSM. Unlike the earlier study, Spanish-speaking MSM prioritized personal interest in a mobile app and de-emphasized the efficiency of an app to make their lives easier in their decision to download an app to their mobile device. Tailoring mobile apps to the language and needs of Spanish-speaking MSM is critical to help increase their willingness to download a mobile app. Despite the growing number of HIV-prevention apps in development, few are tailored to Spanish-speaking MSM, representing an important gap that should be addressed in future research.

## Introduction

### Background

HIV testing is paramount to primary prevention and serves as the gateway into medical care upon receiving a seropositive diagnosis. Compared with other groups in the United States, HIV disproportionately affects gay, bisexual, and other men who have sex with men (MSM). Although the number of new HIV infected results from 2010 through 2014 remained stable among MSM, MSM accounted for 82% of new HIV diagnoses among males and 68% of total new diagnoses in 2015 [1]. If current HIV rates continue, the Centers for Disease Control and Prevention estimates that 1 in 6 MSM will be diagnosed with HIV in their lifetime, which differs by race: 1 in 2 for blacks, 1 in 4 for Latinos, and 1 in 11 for white MSM [2]. Among Latino MSM, diagnoses of HIV increased by 13% from 2010 to 2014, and Latino MSM in the Southern region are more likely to acquire HIV from male-to-male sexual contact than from other routes of transmission in other regions of the United States (eg, primarily intravenous drug use if in the Northeast region) [3]. This body of epidemiological evidence calls for the need to improve HIV prevention efforts for Latino MSM, including testing for HIV and sexually transmitted infections.

Although many MSM test for HIV at least once in their lifetime, opportunities to improve regular HIV testing among Hispanic or Latino MSM are evident. Findings from the 2008 HIV Behavioral Surveillance System found that 63% of Hispanic or Latino MSM had been tested for HIV in the past 12 months, and that recent testing (within the past year) was strongly associated with being of younger age, having seen a health care provider, and disclosure of male-to-male attraction or sexual behavior to a health care provider [4]. Joseph and colleagues reported that among the 608 Hispanic or Latino MSM residing in New York City or Miami, Florida, 31% were test avoiders (those who had never been tested or had last gotten tested for HIV 5 years ago), and 69% received their last HIV test more than 1 year ago. Hispanic or Latino MSM test avoiders were less likely to have incomes over US $10,000 and to have seen a health care provider in the past year [5]. Moreover, in a study with 538 Hispanic or Latino MSM residing in South Florida, Fernandez et al found that 70.4% of men were repeat testers, defined as having been tested for HIV 3 times in their lifetime; men who were repeat testers were more likely to be older, more educated, having a history of diagnosis of sexually transmitted disease, and a greater number of sex partners [6]. Regular testers, defined by receiving 2 tests per year for minimum of 1 year, accounted for 43% of men in the sample; regular testers were more likely to be younger and have lower risk perceptions of acquiring HIV [6]. The aforementioned studies highlight that certain demographics were associated with either being a repeat tester for HIV or an HIV test avoider among Hispanic or Latino MSM in the United States.

One promising area that has garnished increased attention in HIV prevention is mHealth. The World Health Organization defines mHealth as “medical and public health practice supported by mobile devices, such as mobile phones, smartphones, patient monitoring services, personal digital assistants, and other wireless devices” [7].

Smartphones, which include global positioning systems (GPS), text messaging (short message service, SMS), and app features, have become almost ubiquitous among Hispanic adults, including MSM, as they tend to be early adopters of newer technologies [8]. Preliminary work, much of which is qualitative, has begun to understand how best to optimize content, design, and delivery features of mHealth technologies for HIV prevention and testing [9-12]. In the study by Goldenberg and colleagues, MSM preferred their HIV prevention app to (1) have an educational component to guide their decisions for which test is best for them and prevention options, (2) be interactive and engaging with personalized feedback about their own sexual behaviors, (3) provide a social-networking component with other MSM, (4) use language that is simple and understandable to the community, and (5) address privacy concerns by ensuring that the app is from a credible source and having secure messaging features [10]. In the Schnall and colleagues’ study, high-risk MSM described wanting a self-information management system, focus on staying healthy, HIV testing, a chat or communication function, and other related resources in an HIV-prevention app [12]. High-risk MSM in the study by Aliabadi and colleagues stated that they preferred HIV prevention apps to include key information about HIV testing (eg, options) and support group information, motivational messages about addressing sexual encounters in which men intend to use condoms but do not, behavioral skills about negotiating safer sex, and understanding signs of HIV infection [9].

In the study by Mitchell et al, focus groups were conducted with English-speaking MSM (N=34, 68% white, 27% Hispanic) from Minneapolis, Minnesota, and Miami, Florida, to better understand what features and functions of a mobile app they believe are related to whether they download and continue to stay engaged with the app on their mobile device [11]. Men stated that they tend to download apps that they perceive to be low cost (eg, free), secure, and efficient in helping them save time and for adding convenience to their lives [11]. Features and functions that increased men’s willingness to remain engaged with apps on their mobile device over time (some of which were also related to downloading the app initially) included how useful and necessary the app was to them, reviews from other users and recommendations from friends, ease of use, how reliable the app was, and how frequently the app was updated [11]. When asked about their preferences for an HIV-testing app, men stated that they would like an app that contained a tailored (to their sexual activity) testing interval recommendation, an HIV-test locator, and a way to monitor their personal sexual behaviors [11].

### Objective

The studies described above provide a growing body of literature to inform new mHealth interventions; however, these studies primarily focus on English-speaking white, black, and MSM of other races. To our knowledge, no studies have asked primarily Spanish-speaking MSM in the United States about their app use, attitudes, and motivations for downloading and sustaining use of mobile apps, and preferences with respect to HIV prevention. Building on previously published work (eg, [11]), we conducted individual interviews with Spanish-speaking, HIV-negative MSM to explore what features and functions of mobile apps that participants believed were related to downloading and continuing to use apps on their mobile device, which could then be applied toward development of an app to promoting regular HIV testing. We recruited Hispanic, Spanish-speaking MSM living in Miami, Florida, to assess whether findings from previous work align with this group of men living in a different area of the United States. The primary objectives of this study were to answer the following questions:

1. What features and functions of smartphone apps do Hispanic, Spanish-speaking MSM believe are associated with downloading apps to their smartphones?
2. What features and functions of smartphone apps are most likely to influence mens’ sustained use of apps over time?
3. What features and functions do men prefer in a smartphone app aimed to promote regular testing for HIV?

## Methods

### Recruitment and Eligibility

Recruitment for the study sample was conducted through targeted Facebook advertising, flyers displayed in places where MSM frequent, and through referrals as a form of snowball sampling.

Facebook advertisements were conducted in early 2016 and targeted individuals who described themselves as Spanish or bilingual (Spanish- and English- speaking) males who were aged at least 18 years, living in the metro area of Miami, Florida, and interested in men. Each targeted advertisement contained a picture of a Hispanic male with a brief study description that stated participation included a confidential, individual interview with a link to the eligibility screener. Each of the 2 campaigns lasted 3 days with advertisements appearing on the home page, desktop newsfeed, and mobile newsfeed of persons whose Facebook profiles met the targeting criteria.

Study flyers were displayed at establishments that MSM frequent (eg, bars and nightclubs) and at community-based organizations and health clinics that provide HIV testing in Miami, Florida metro area. Flyers contained a brief description of the study with a weblink for the eligibility screener. In addition, participants who were eligible and enrolled in the study were asked to share information about the study with their Spanish-speaking MSM peers.

The targeted Facebook advertisements generated 2131 Web clicks to the eligibility screener, which resulted in 106 individuals completing the eligibility screener. Out of the 15 participants (14.2%) that were eligible and provided consent to participate in the study, 5 learned about the study from Facebook advertisements, 9 were referred by other participants, and 1 participant was recruited via a flyer.

To participate, individuals had to have met the following self-reported eligibility criteria: (1) identify as male, (2) be 18 years of age or older, (3) live in the metro area of Miami, Florida, (4) be HIV-negative or have an unknown serostatus, (5) be a Spanish or bilingual (Spanish and English) speaker, (6) have had anal sex with another man within the past year, and (7) be a current owner of a smartphone. Once deemed eligible via the online screener, participants were asked to provide consent electronically and provide 2 contact methods so that the research team could coordinate and schedule their interviews.

### Interviews

After providing consent and contact information, participants were contacted by the study team to schedule an appointment for their one-time, in-person, confidential interview. All interviews were conducted in Spanish, audio recorded, transcribed verbatim in Spanish, translated to English, checked for accuracy, and then de-identified for anonymity purposes. The interview was semistructured, focusing on Spanish-speaking MSM’s smartphone preferences (eg, likes, dislikes), apps they currently have and use on their smartphone, and their reasons for downloading and keeping apps on their smartphone over time—defined as 3 months or longer. In addition, participants were asked their opinions about health-related apps and suggestions to design a smartphone app for MSM to encourage regular testing for HIV. Finally, participants were asked to provide feedback on potential features of an HIV-testing app, such as informational messages, testing interval developer, monitoring their sexual activities, and a GPS function to locate nearby HIV-testing locations. Upon completion of the interview, each participant received an incentive of US $50 for his time.

### Data Analysis

Content analysis and grounded theory were used to analyze the data. A team of 2 independent research associates coded data from the interviews. One of the independent research associates was formally trained in qualitative methods and had several years of experience as a qualitative researcher. This individual led several meetings to (1) train the other associate in qualitative coding before the coding process began and (2) to facilitate discussions and resolve disagreements regarding coding differences, after each associate coded the interviews.

Both inductive [13] and deductive processes were used as each associate independently coded the interview transcripts and created a codebook in a 3-step process:

1. Each associate independently read all interviews and identified a preliminary set of codes or items found in the interviews (an inductive process). These emerging codes were statements or words that referred to events, behaviors, or activities that occurred frequently or rarely, and which were similar and/or different to other codes in the transcript.
2. Following the grounded theory approach, each associate independently clustered or grouped identified codes or items that fit together into larger categories of meaning (themes or patterns) and those who did not fit any theme (a deductive process) [14]. In doing so, associates paid specific attention to the contexts and behaviors of the phenomena under study as emerging from interviewees’ statements.
3. With this initial-coding scheme (or codebook), each associate recoded the data using the identified themes or patterns. During this process, each associate refined, added, and/or eliminated codes and themes to ensure all possible meanings were accurately captured. A spreadsheet was used to organize codes under each pattern or theme and all quotes associated with each code.

The 2 associates met to review the coding spreadsheets (codebooks) that emerged from the individual process mentioned above. They first looked at agreements. Agreement implied that both associates had identified the same codes or themes or patterns from the same interview quotes. Then, disagreements were discussed. Disagreements ranged from using different labels to name a code or a pattern, to finding that one coder identified a code or a pattern but the other associate did not. In those cases, both associates collectively made a decision as to how the disagreement would be resolved. In those few instances, associates collectively modified codes where inconsistencies were found and ensured all possible meanings were fully and accurately captured. Finally, a master Excel file with an identical codebook was created to reflect the agreements reached by both research associates, which they both then used and applied across all interviews.

## Results

### Characteristics of the Sample

Table 1 provides sociodemographic characteristics of the study sample. On average, men were 32.4 years old; their ages ranged from 18 to 68 years. All men ethnically self-identified as Hispanic; with respect to race, 10 of the men were self-reported as white, 1 as black, and 4 as another race. Regarding HIV testing, 7 men tested 1 to 3 months before study enrollment and 2 other men had gotten tested 4 to 6 months before the study. The timeframe of when the remaining participants had last gotten tested for HIV varied; 2 men reported that they had never been tested for HIV. Table 2 provides information about the themes and associated definitions for participants’ reasons to download and continue the use of apps on their smartphone, as well as their preferences for an HIV testing app.

**Table 1:** Sociodemographic characteristics of the study sample.

Characteristic		Value
Total number of participants, N		15
Age in years, mean (range)		32.4 (20-68)
**Race, n (%)**		
	White	10 (67)
	Other	4 (27)
	Black	1 (7)
**Ethnicity, n (%)**		
	Hispanic	15 (100)
**Most recent HIV test, n (%)**		
	1-3 months ago	7 (47)
	4-6 months ago	2 (13)
	7-9 months ago	1 (7)
	10-12 months ago	1 (7)
	More than 1 year ago	1 (7)
	5 or more years ago	1 (7)
	Never been tested	2 (13)
**Type of smartphone owned, n (%)**		
	iOS	8 (53)
	Android	6 (40)
	Windows	1 (7)

**Table 2:** Themes, definitions, and participants’ general reasons to download and continue the use of apps and their preferences for an HIV testing app.

Category	Theme	Definition
Reasons for downloading an app	Personal interest	Downloading the app because it is of personal interest.
Reasons for downloading and using the app over time	Keeping personal information secure	The app being secure in terms of access to and/or protecting personal information.
	Cost	Download an app if it requires an initial payment or keep using an app if it requires additional payments.
	Influence by peers and posted reviews	Download and/or continue to use a certain app because of positive reviews by peers, friends, or app ratings.
	Ease of use	Downloading and sustaining use of an app because of the ease of use.
	Functionality	Discuss the importance of the app being functional and fulfilling a specific need.
Reasons for keeping and using the app over time	Entertainment	Discussed importance of enjoying the app to continue its use.
	Updates	Frequency in which an app is updated.
Preferences for HIV testing app features and functionality	Informational messages	Discussed opinions about receiving informational messages about the importance of testing for HIV and other sexually transmitted diseases.
	Monitor sexual activity	Opinions shared about monitoring their own sexual behaviors.
	Recommended testing intervals with dates	Discussed receiving personalized, recommended testing intervals with specific dates of when to be tested next.
	Details about testing locations and HIV test locator	Opinions shared about wanting to know nearby locations to test and information about the testing sites.

### Reasons to Download an App

Personal interest in an app was the theme discussed as a reason for downloading an app. More than a quarter of participants shared that the app should be of personal interest in order to download it:

If it is of my interest I download it.

49 years old, white, last HIV test 1-3 months before study

It has to be something that interests me. Anything, but that it interests me.

38 years old, other race, last HIV test was 5 or more years before the study

### Reasons to Download and Use the App Over Time

Interviews offered several reasons for downloading and using the app over time (ie, 3 months or longer), such as security of personal information, the cost of the app, influence from reviews and peers, the ease of use, and its functionality.

#### Keeping Personal Information Secure

All participants agreed that to download an app, it must be secure at keeping their personal information confidential and private. As this participant aptly stated:

...that’s 100% important, because if it is not safe, what sense does it make to download it?

42 years old, black, last HIV test was 1 to 3 months before the study

Men also perceived security to be an important factor that influenced them to download and use an app over time, particularly with respect to the types of data they were comfortable sharing with the app:

I care a lot about that...only hotels and airlines and that’s it. The rest I do not put my credit card or my personal information anywhere.

38 years old, other race, last HIV test 5 or more years before the study

#### Cost

The majority of the participants (n=12) stated that they were willing to pay to download an app or pay a monthly subscription fee as long as the app is useful to them:

It depends on the application. For example, if it is necessary to use, I cannot live without it, of course. But for example, I would not pay to download a social network on my mobile phone.

21 years old, white, last HIV test was 1 to 3 months before the study

Another participant stated:

Depending on how good is the application and how useful it is; I consider on paying monthly subscription.

23 years old, Other, never been tested for HIV

Fewer men (n=3) expressed that they were not willing to pay to use an app and chose to only download free apps:

Most of the time they don’t have a cost, because you look for free applications...I never, when they start to ask information about a card I leave it there.

49 years old, white, last HIV test was 1 to 3 months before the study

Another interviewee stated a free trial is a must:

I think it's the best way. I think the best way because one really sees if it works or does not work...it happened to me that I downloaded applications, and then paid five dollars and it does not work.

30 years old, white, last HIV test was 1 to 3 months before the study

#### Influence by Peers and Posted Reviews

Participants were influenced to download an app by the reviews that came from friends or word of mouth:

That is, if all my friends have and say, “Hey download it, is good,” obviously I am inclined to download

27 years old, other race, last HIV test was 1 to 3 months before the study

Other participants were influenced by the app ratings:

See the reviews, reports that people have about the application and if it is good or bad and all that.

23 years old, other race, never been tested for HIV

Few participants mentioned their influence to download an app came from the media or social media:

Well, if I see an application on TV.

49 years old, white, last HIV test was 1 to 3 months before the study

Five participants identified that friends using the app was important for them to keep using the app over time:

If they have it yes and if it is good...

35 years old, other race, last HIV test was 7 to 9 months before the study

Other 3 participants partially agree with the statement above:

Yes, medium, if my friends leave the application, but I still made me useful, I keep it.

23 years old, other race, never been tested for HIV

Six participants claimed that friends using the app had no influence in them keeping an app:

No, if the need does not matter that they do not use it and I do. It does not matter. It is not a priority for me.

42 years old, black, last HIV test was 1 to 3 months before the study

#### Ease of Use

Besides security and cost, the majority of interviewees identified the importance of intuitive features and functions to download an app:

If I download it [app], it has a friendly interface and is not very difficult to use.

30 years old, white, last HIV test was 1 to 3 months before the study

Another participant stated:

...and one that is also easy to handle, because if it is too complicated, I do not care. So basically, that works for me and easy to navigate and use.

42 years old, white, last HIV test was 1 to 3 months before the study

Ease of use also influenced men on whether they kept using the app over time:

...easy to navigate within the application or not. If it is too complicated, like it doesn’t go...

20 years old, white, never been tested before

Another participant said:

Very important because...I handle the Smartphone to its fullest, but for others it becomes complicated perhaps...you have to have an easy interface.

30 years old, white, last HIV test was 1 to 3 months before the study

#### Functionality

Participants also indicated that the app needed to be functional and to help fill a particular need of theirs in order for them to download it and keep using an app over time. This participant aptly expressed the importance that function and perceived need for the app influence their desire to download it:

First the functionality of the application, if it’s really going to work for me, for what I want, for what I need.

30 years old, white, last HIV test was 1 to 3 months before the study

Another man in the group expressed how functionality of the app promotes continued use of the app over time:

The main reason is it useful to me, as I had said earlier, the utility that has the application and I would say would be the main reason to keep an application that is useful to me. The usefulness of the application.

42 years old, white, last HIV test was 1 to 3 months before the study

### Reasons for Using the App Over Time

Themes solely related to mens’ reasons for using an app over time (ie, 3 months or longer) included entertainment and app updates.

#### Entertainment

More than half of the interviewees (8) explained that they used an app over time if it provided them with some form of entertainment:

The truth is that if keeps me entertained or I get notifications, such as news and all that, then yes, if not, I delete it.

23 years old, other race, never been tested for HIV

Another participant stated:

If entertains me, if I find useful, if I finished- is, I don’t know. That is, if I end up liking, obviously I will continue using and all that. So I think that, if entertains me.

20 years old, white, never been tested for HIV

#### Updates

In addition to entertainment, only 3 interviewees mentioned the importance of frequent updates for using the app over time:

If there are constant updates, all that. I find it important that they are constantly updated, to do new things, new features, all that.

20 years old, white, never been tested for HIV

Another participant said:

Do not get stuck much. That they are always doing updates...you have something new.

20 years old, white, last HIV test was 10 to 12 months before the study

### Reasons for Deleting an App

There were 4 reasons why participants chose to delete an app—dislike, lack of use, cost, and lack of memory or space. Several participants mentioned dislike (n=4) and lack of use (n=4) as major reasons for deleting an app:

It depends. That is, if it is something of continuous necessity, or continuous use, I keep using it...and if I no longer need it, I delete it, so it does not take up space on the phone.

27 years old, other race, last HIV test was 1 to 3 months before the study

Another participant stated:

I try it first, if I don’t like it maybe in the moment I de-install...two or three applications per month that I didn’t like, I discard them.

49 years old, white, last HIV test was 1 to 3 months before the study

Likewise, a few interviewees (n=4) stated cost and lack of memory as reasons for deleting an app:

I eliminate them...if they ask for a price I can’t afford.

68 years old, white, last HIV test was 1 to 3 months before the study

Another participant said:

Every time I run out of memory, I decide to delete applications that have not use in a long time.

21 years old, white, last HIV test was 1 to 3 months before the study

### Preferences for HIV Testing App Features and Functionality

The final part of the interview explored participant’s attitudes toward potential features of a hypothetical HIV-testing app. The specific features explored included, informational messages, sexual activity monitor, recommended testing interval with dates, details about testing locations, and an HIV-testing locator.

#### Informational Messages

The majority of interviewees would like to receive HIV testing interval messages. The discussion of the informational messages centered around 2 primary components—frequency and format. For frequency, some participants discussed that they would like to receive informational messages once a week:

Well, weekly...It's fine, because that is, as I said before helps you keep in touch with yourself, so to speak. Your health is also important because it is not - is not a myth that health always left aside.

25 years old, white, last HIV test was 4 to 6 months before the study

Others would like extended time intervals between these messages:

Once every two weeks.

27 years old, other race, last HIV test was 1 to 3 months before the study

Another participant stated:

Once a month for you to be aware of how you can take care of yourself...

35 years old, other race, last HIV test was 7 to 9 months before the study

For format, some participants would rather receive the message through the app:

It can be like an alert if you have the application, yes, like a notification. I do not - in my case it would have no problem. But a notification seems very well to me.

25 years old, white, last HIV test more than a year before the study

Others would rather receive the informational message via email or text:

I think through text is cool, super cool. Because you know there are people who sometimes do not like notifications that comes up on their screen...And I think by text or email, for me it would be fine.

20 years old, white, last HIV test was 10 to 12 months before the study

#### Sexual Activity Monitor

In addition to receiving test interval messages, most participants agreed with the idea of including a sexual monitor feature in the app:

I think it's fun that you can realize: “Oh, this is the amount of times I've had sex this week.” “This is the amount of times I've drink-.” Then, like: “Can I do better?...That is: “Can I stop? Can I take more care of myself?”

21 years old, white, last HIV test was 4 to 6 months before the study

Another participant stated:

Yes, that is as the application has to do with that part of what- to make checkups regularly- I think it's okay to keep [track of] your activity, therefore, that, you know, if you had relations with six people this week is like well, you know you have to make a checkup.

20 years old, white, never been tested before

In contrast, one participant indicated that he did not see the purpose of this sexual activity monitor:

I do not see the point. I mean, why would someone do something like that? I mean, I do not see what could be the purpose of that. I mean, I would not see the usefulness.

38 years old, other race, last HIV test 5 or more years before the study

#### Recommended Testing Intervals With Dates

Although 3 men did not provide an opinion, all other participants universally liked the recommended testing interval as an app feature. Many of the participants succinctly shared their support for this idea by saying:

It sounds good. Sounds good to me.

25 years old, white, last HIV test more than a year before the study

In addition to supporting and liking this idea, other participants provided more context as to why this idea of being provided with recommended HIV-testing intervals with dates appealed to them:

In the event that someone will become interested, I think is super good, because if you are giving it as some guidance. That's what you were talking about a guide, it is very clear. If that can send with a hidden notification, great.

38 years old, other race, last HIV test 5 or more years before the study

#### Details About Testing Locations and HIV-Testing Locator

Another feature with unanimous consensus among participants was the inclusion of GPS function that would allow them to find the nearest testing center should be included in the app:

Very useful, because there is often the laziness factor and time factor as to the availability of people, not often to use them. And if you have something that reports what is closest to you and that will save time, you will save money, then so be it.

25 years old, white, last HIV test was 4 to 6 months before the study

And another participant stated:

Fabulous, because see I was about to do it a while ago and I couldn’t find a place.

49 years old, white, last HIV test was 1 to 3 months before the study

## Discussion

**Principal Findings**

A growing body of literature has examined MSM’s preferences for app design, features, and functionality to direct the development of mobile apps addressing a variety of HIV prevention outcomes (eg, HIV testing, reduce engagement in condomless anal sex) [10,15]. However, to our knowledge, this is the first study to explore men’s preferences in a sample of Spanish-speaking MSM, who are important targets for HIV prevention services [2]. Overall, this study showed that Spanish-speaking MSM reported many of the same considerations (eg, ease of use, perceived usefulness, security) that impacted their adoption and use of mobile apps and other technologies as shown in prior studies of primarily English-speaking MSM [11,15]. In addition, Spanish-speaking MSM noted that having a strong personal interest in the content of the mobile app strengthened their motivation to download the app on their phone. These results are discussed in more detail below.

Prior work has shown that English-speaking MSM in Minneapolis, Minnesota, and Miami, Florida, who participated in focus groups considered cost, security, and efficiencies afforded by the app in their decision to download it, whereas influence by others (either through reviews or friends) and the app’s usefulness was related to both downloading and sustaining use (Table 3) [11]. Continuing to use apps on their mobile device was primarily attributed to how easy the app was to use, the reliability of the app, and how frequently it was updated. Compared with these findings, Spanish-speaking MSM also endorsed the importance of these factors but believed that many of them (ie, cost, security, influence by reviews or friends, usefulness, and ease of use) contributed to their willingness to both download the app and continue using it (Table 3). Spanish-speaking MSM did not identify an app’s ability to make their life more efficient as a primary motivator to downloading apps to their mobile device; however, they did state that a strong personal interest in an app was important to motivate them to download the app. This finding brings to light the importance of developing tailored HIV prevention apps for Spanish-speaking MSM that address their unique values and preferences (both their language preferences and preferences for content and features) in order to increase their personal interest in the app and motivate them to download it to their phone.

A number of in-person [16-19] and computer-mediated [20] intervention approaches have been tailored to the needs of Spanish-speaking MSM. However, to our knowledge, no Spanish-language HIV prevention mobile apps have been developed and tested for Spanish-speaking MSM in the United States. These finding suggest that this may be an important avenue for future research on using mobile apps to deliver HIV prevention interventions to this group.

Men in this study were also asked to reflect on their preferences for features of an HIV-testing mobile app. There was variation among men in this sample for the frequency and delivery method for informational messages (eg, about risk of HIV or the importance of HIV testing). Some men wanted informational messages to be sent through the app (ie, as a notification), whereas other men preferred an SMS (text) message. Some men preferred weekly informational messages, whereas other men expressed the desire for less frequent messages. Together, this suggests that giving Spanish-speaking MSM options for frequency and format of informational messages may be the most effective way to accommodate their varied preferences for these factors.

**Table 3:** Comparison of themes related to downloading, downloading and sustained use, and sustained use of smartphone apps between English-speaking men who have sex with men (Mitchell et al, 2016) and Spanish-speaking men who have sex with men (MSM).

Reason	Downloading		Downloading and sustained use		Sustained use	
	English	Spanish	English	Spanish	English	Spanish
Cost	✓			✓		
Security	✓			✓		
Efficiency	✓					
Personal interest		✓				
Influence from reviews or friends			✓	✓		
Usefulness or functionality			✓	✓		
Ease of use				✓	✓	
Reliability					✓	
Updates					✓	✓
Entertainment						✓

In contrast, there was nearly universal or universal support for mobile app features that included an HIV-test locator (100% support), a recommended HIV test interval period for men to get tested (80% support), and to monitor their sexual activity (93% support). English-speaking MSM in a prior study also unanimously supported an HIV-test locator and a recommendation for the best date range to be tested for HIV; however, approximately three-fourths supported a feature for monitoring their sexual behaviors [11]. Differences in support for this feature may be due to different beliefs about the usefulness of sexual activity monitoring, or concerns about the security of storing sexual data on their mobile device. Both Spanish- and English-speaking MSM stated that security considerations were important to them [11]; perhaps suggesting the perceived usefulness of this feature may be the primary reason to explain differences in their support for sexual activity monitoring. A separate study also reported some support among English-speaking MSM residing in Atlanta, Seattle, and rural areas of the United States for monitoring sexual activity and feedback on high-risk encounters [10]. Clearly, continued research is needed to more fully understand MSM’s comfort level with sexual self-monitoring and how best to include this feature for men who may be culturally unique.

### Limitations

This study has important limitations to acknowledge. First, the results from these interviews are not intended to be generalizable to all Spanish-speaking, HIV-negative MSM who are smartphone owners in the United States. Participants in this study resided in one large urban area (Miami, Florida) and, as such, app preferences may differ from Spanish-speaking MSM residing in different regions of the United States. Future research should assess smartphone app preferences among a more geographically diverse sample of Spanish-speaking MSM to determine whether similar attitudes are expressed. Future studies should also consider whether general smartphone app preferences and those related to HIV prevention and testing differ between bilingual MSM and monolingual Spanish-speaking MSM. Second, recall bias may have affected participants’ abilities to accurately identify features and functions that influenced them to download and use apps over time. The qualitative, cross-sectional nature of this study prohibits any causal inference, and longitudinal studies are needed to confirm the findings of this study. These results are meant to be first steps in more fully understanding the needs of Spanish-speaking HIV-negative MSM with respect to mobile app preferences for HIV prevention.

### Conclusions

This study presents findings of formative work to understand which features and functions of mobile apps that Spanish-speaking HIV-negative MSM perceived as important in their consideration to download mobile apps to their phone and continue to use them over time. Men also provided critical feedback on features of an HIV-testing app they believed are important to incorporate in a future mobile app. Overall, Spanish-speaking MSM endorsed many of the same priorities expressed among English-speaking men in prior studies [10,11,15]. However, men in this study showed high support for monitoring their sexual activities using a mobile app and believed that personal interest was a strong influence on their willingness to download an app to their mobile device. These findings lend support to developing tailored HIV prevention apps for Spanish-speaking MSM, which is a notable gap in current prevention efforts. These findings may help guide future efforts in developing an HIV prevention app for Spanish-speaking MSM, although more research is needed to fully understand how to best frame content in the app and how to reach Spanish-speaking MSM with mobile app interventions. Furthermore, other research with larger samples of Spanish-speaking MSM is warranted to fully address these gaps in knowledge and whether cultural appropriateness plays a role in future development of HIV prevention apps, including those aimed to promote regular HIV testing.

## Abbreviations

GPS: global positioning systems
MSM: men who have sex with men
SMS: short messaging services
